# Therapeutic potential of an anti-CCR9 mAb evidenced in xenografts of human CCR9^+^ tumors

**DOI:** 10.3389/fimmu.2022.825635

**Published:** 2022-07-27

**Authors:** Silvia Santamaria, Marisa Delgado, Marta Botas, Eva Castellano, Isabel Corraliza-Gorjon, Paloma Lafuente, Cecilia Muñoz-Calleja, Maria L. Toribio, Leonor Kremer, Jose A. Garcia-Sanz

**Affiliations:** ^1^Centro de Investigaciones Biologicas Margarita Salas (CIB-CSIC), Department of Molecular Medicine, Consejo Superior de Investigaciones Científicas, Madrid, Spain; ^2^Centro Nacional de Biotecnología (CNB-CSIC), Department of Immunology and Oncology, Consejo Superior de Investigaciones Científicas, Madrid, Spain; ^3^Servicio de Inmunología, Instituto de Investigación Sanitaria Hospital Universitario de La Princesa, Universidad Autónoma de Madrid, Madrid, Spain; ^4^Centro de Biología Molecular Severo Ochoa (CBMSO-CSIC-UAM), Consejo Superior de Investigaciones Científicas, Madrid, Spain

**Keywords:** chemokine receptor CCR9, antitumoral activity, combined chemotherapy and immunotherapy, orthotopic xenograft mouse model, therapeutic antibodies, T-ALL leukemia, CCR9 positive T-ALL leukemia

## Abstract

Relapsed or refractory T acute lymphoblastic leukemia (T-ALL) still carries poor prognosis. Aiming to improve outcomes, the therapeutic potential of an anti-CCR9 monoclonal antibody (mAb 92R), targeting the human chemokine-receptor CCR9 is analyzed on orthotopic xenotransplants. 92R mAb treatment of mice carrying human CCR9^+^ T-ALL cell lines or primary T cell leukemias inhibits tumor growth and increases survival. The therapeutic effects of 92R are specific and synergize with chemotherapeutic agents increasing survival. Furthermore, 92R decreases size of non-hematopoietic tumors with a forced CCR9 expression and of solid tumors generated by the pancreatic adenocarcinoma cell line AsPC-1. In addition, a humanized version of 92R mAb (Srb1) is also able to inhibit growth of CCR9^+^ T-ALL tumor cells *in vivo*, increasing survival 2.66-fold. Finally, 92R mAb prevents liver accumulation of infiltrates and reduces tumor cell numbers in already formed infiltrates. Thus, the humanized version of 92R mAb (Srb1), displays therapeutic potential for CCR9^+^ tumor treatment and might represent one of the first therapeutic antibodies for precision medicine on T-ALL patients.

## 1 Introduction

Chemokine receptor signaling, after interaction with their ligands, is crucial for lymphocyte trafficking and organogenesis, both in homeostasis and inflammation (1). In cancer, a strong association between aberrant chemokine receptor expression on tumor cells with cancer progression, poor prognosis and organ selective metastases has been described (2–4). CCR9 is a G-protein-coupled 7-transmembrane receptor protein that belongs to the chemokine receptor family. Its expression in normal cells is restricted to immature thymocytes (5–8), immune cells infiltrating the small bowel (9), 3% of the circulating memory α4β7^high^ T lymphocytes (10), IgA secreting plasma B cells (1) and plasmacytoid dendritic cells (11). The chemokine CCL25 (formerly TECK), is the only known ligand for CCR9 (6, 12). This chemokine is secreted by epithelial and dendritic cells in the thymus (12, 13) and by small intestine crypt epithelial cells (9). Thus, interaction of CCR9^+^ cells with CCL25 directs their migration mainly to the small intestine.

The data on CCR9 expression in tumor cells, although scarce, allowed to demonstrate a positive correlation between CCR9 expression and the tumor ability to generate metastases in the small intestine (14–16), as well as between CCR9 over-expression and disease aggressiveness in acute and chronic T cell leukemias (14). In addition, aberrant CCR9 expression in melanoma, prostate, breast or pancreatic tumors, has been correlated with *in vitro* invasiveness in response to CCL25 (14, 16–23). Furthermore, expression of CCR9 on tumor cells leads to competitive advantages for these cells, since CCL25 engagement enhances cell survival and prevents apoptosis *via* the phosphatidylinositide 3-kinase (PI3K)/Akt pathway on several solid tumors (20, 21, 24–30); it also activates the JNK1 anti-apoptotic pathway in leukemic cells (31) and participates in Notch1-mediated cell proliferation (18).

92R is a mouse mAb that recognizes the human CCR9 receptor and inhibits the growth of subcutaneous MOLT4 (CCR9^+^) cell xenotransplants in immunocompromised animals (32). To determine the robustness of 92R mAb as a potential therapeutic agent for tumor growth, here we analyze the effects of this mAb on orthotopic xenotransplants in NSG animals. Using this approach, it could be demonstrated that 92R mAb, and its humanized version (Srb1), both induce a strong specific inhibition of tumor growth *in vivo*, increasing survival of the xenotransplanted animals around 2.6-fold, as compared to isotype control mAb treated animals. In addition, 92R mAb also increased survival in xenotransplanted animals carrying primary CCR9^+^ T cell leukemias and decreased the number of tumor-carrying animals, and their tumor size, on subcutaneous non-hematopoietic xenotransplants expressing CCR9. Furthermore, since the mechanism of action of Srb1 and chemotherapeutic drugs are different, they synergize increasing survival of the xenotransplanted animals. Finally, 92R and Srb1 also exert their function on infiltrating cells, preventing on the one side liver infiltration, and on the other, decreasing the tumor cell numbers on already formed infiltrates. Taken together, these data suggest that the humanized version of 92R mAb (Srb1), display a strong therapeutic potential for the treatment of CCR9^+^ leukemias and other CCR9^+^ tumor types.

## 2 Material and methods

### 2.1 Cells and reagents

The human T-ALL cell lines MOLT-4 (CRL-1582) and Jurkat (TIB-152), and the human embryonic kidney cell line HEK293T (CRL-3216) were obtained from the American Type Culture Collection (ATCC). MOLT4 and Jurkat cells were cultured in Dulbecco’s modified Eagle’s media (DMEM) supplemented with 10% heat inactivated (30 min, 37°C) fetal bovine serum (FBS), 2 mM L-glutamine, 0.1 mM non-essential amino acids, 10 mM Hepes (pH 7.0) and 1 mM Na-Pyruvate (media and supplements all from Gibco). MOLT4-GFP and Jurkat-GFP were obtained by electroporation (4x10^6^ cells) in 400 µl of complete media with 5 µg of a pEF1α^prom^-eGFP plasmid. Cells were selected for 2 weeks in 1 mg/ml G418 (Gibco) and subsequently sorted to retain the highly GFP^+^-cells. MOLT-4 cells carrying a luciferase expression vector (MOLT-4-luc) have been previously published [33]. The HEK293T cells were cultured in DMEM supplemented with 10% heat inactivated FBS and 2 mM L-glutamine. These cells were transfected using XtremeGene9 DNA transfection Reagent (Roche), with a hCCR9-GFP cDNA inserted in an expressing plasmid under the control of EF1α promoter. The GFP-expressing transfectants were selected by FACS sorting. The AsPC-1 cell line (ATCC, CRL-1682) is derived from xenotransplants carried out in nude mice with the ascites of a 62 years-old white female diagnosed of a pancreatic adenocarcinoma (33).

The following antibodies were used: 92R mouse mAb anti-hCCR9 (IgG2a) [32], isotype control mouse antibodies (IgG2a and IgG2b) [32]. The humanized version of 92R mAb (Srb1) was generated by inserting the CDR sequences of 92R (CD1, CDR2 and CDR3) from the heavy and light chains into human germ line framework sequences for IGH and IGK genes, which were subsequently fused to the constant IgG1/k immunoglobulin regions, and then cloned on the pEE (Lonza) expression vector. The secreted single chain antibody, obtained from transient transfection of HEK293 cells was purified on Protein A Sepharose and verified by SDS-PAGE under reducing conditions. Srb1 had K_D_ comparable to 92R. The Srb1 antibody and its isotype control (human IgG1 control, #C0001-5, CrownBio, San Diego, USA) used for these experiments were kindly provided by SunRock Biopharma.

#### 2.1.1 Blood samples from T-ALL patients

The experiments reported here were approved by the CSIC Ethics Committee. The blood samples from T-ALL were obtained from patients that had signed an informed consent.

Peripheral blood from two T-ALL patients at diagnosis was used to determine the expression of CCR9 by flow cytometry. Patient #1, 47 years-old male, it had 7% of blasts in peripheral blood and 32% in the bone marrow aspirate, the patient showed the following mutations: Gene IDH2 c.419G>A; Gene KRAS c.182A>C; Gene BCORL1. c.4260_4261insGGCTTCC; Gene RUNX1. c.179C>T; Gene ETV6. c.602T>C. Four days before the blood extraction the patient started treatment with methyl-prednisolone. Patient #2 is a 29 years-old male, diagnosed of ALL pro-T, it had 91% of blasts in peripheral blood and 95% on the bone marrow aspirate. The karyotype shows 47,XY,+4[29]/47,XY,+4,del(6q)[2]/46,XY[6] indicative of two related clonal lines and the cytogenetics shows t(14q11)/a TCRα/δ rearrangement. The patient showed the following mutations: Gene JAK3. c.1533G>C; Gene PHF6. c.585T>A; Gene SH2B3. c.1022-2A>G y c.1286T>.

The blood samples were diluted in 3 volumes of 1x PBS-EDTA, layered on a Ficoll-Paque (Pharmacia) step gradient and after centrifugation at 400 x g for 30 min at room temperature, without brake, the peripheral blood mononuclear cells were recovered from the interphase. The cells were washed twice with 1x PBS-EDTA (250 x g, 5 min, room temperature), counted on a CASY-TTC counter and stained with 92R or an isotype control monoclonal antibody and analyzed by flow cytometry.

#### 2.1.2 Primary tumor samples

The experiments reported here were approved by the CSIC Ethics Committee. The human primary T-ALL were obtained from patients that had signed an informed consent.

HLPR primary cells were obtained from a 31-years old patient at diagnosis, initially characterized as CD34^+^, 30% CD3^+^, CD7^+^, 30% CD5^+^, 60% Tdt^+^, CD2^-^, 5% CD1α^+^, TCRαβ^-^, TCRγδ^-^, cytTCRβ^-^, CD4^-^, CD8^-^, CD10^-^, CD56^-^, CD16^-^, 50% CD45RA^+^, CD99^+^, HLA-DR^-^, CD38^+^, CD71^+^, CD33^+^, 18% CD13^+^, and negative for myeloid markers and were diagnosed as early T-cell precursor lymphoblastic leukemia (WHO 2016). These cells are CCR9^+^ and the 85% of them express 3.6-fold less CCR9 in their cell surface than MOLT4 cells. The cells were maintained as intravenous xenotransplants in NSG mice.

T-ALL18 primary cells were obtained from a patient diagnosed of T-ALL leukemia and initially characterized as 50% TCR^+^, 35% pre-TCR^+^, IL7R^+^, CXCR4^+^, and 40% CCR9^+^. These cells were maintained as intravenous xenotransplants in NSG mice. T-ALL18 cells are CCR9^+^ expressing 17.5-fold less CCR9 in their cell surface than MOLT4 cells.

T-ALL10 primary cells were obtained from a patient diagnosed of T-ALL leukemia and initially characterized as TCRαβ^-^, TCRγδ^-^, CD5^+^, CD7^+^, CD4^+^, CD8^-^, IL7R^+^, CXCR4^+^, CCR9^+^. These cells were maintained as intravenous xenotransplants in NSG mice. Currently, T-ALL10 cells express very low levels of CCR9.

### 2.2 Flow cytometry

For staining, 2 x 10^5^ cells/well were centrifuged in 96-well plates and washed with phosphate-buffered saline, pH 7.4 (PBS) supplemented with 1% bovine serum albumin (BSA), 1 mM EDTA and 0.09% sodium azide (wash buffer). Non-specific binding of the mAb to the cell surface was blocked by pre-incubating the cells with 10% (v/v) goat serum in a 100 μl final volume (20 min, 4°C). Cells were incubated with the primary mAb (30 min, 4°C), washed and the binding was revealed with an AlexaFluor488-, AlexaFluor647- or PE-goat F(ab’)2 anti-mouse IgG (H+L) secondary antibody (Life Technologies; 30 min, 4°C). After two washes, samples were analyzed on an Epics XL or a FC500 cytometer (Beckman Coulter). GFP expressing cells on the organs of animals injected with MOLT4-GFP or Jurkat-GFP, were evaluated by flow cytometry on single cell suspensions of these organs.

### 2.3 Xenograft assays

The animal experiments were approved by the Agriculture Department from the Community of Madrid with the references PROEX 038/17 (to JAGS) and PROEX 164/16 (to LK). The animal housing, breeding and experiments were carried out in adherence to the NIH “Guide for the Care and Use of Laboratory Animals” and in accordance with EU and Spanish laws.

NOD.Cg-Prkdc^scid^ Il2Rγ^tm1Wjl^/SzJ stock #005557 (NSG mice, Jackson Laboratories, Maine, USA) were bred at the CIB and CNB animal facilities. For orthotopic xenotransplants, on day 0 animals were injected on the tail vein with the appropriate tumor cells (1x10^6^). The animals were divided into several groups that were treated intraperitoneally (i.p.) with 4 mg/kg of 92R mAb (anti-hCCR9), isotype control (IgG2a), or 100 µl PBS, on schedules described for each experiment. In principle, the animals received the first dose during the first week after injection of the tumor cells (days 2-7) and a second dose 7 days after the first. In some occasions, when the tumors developed with a slow kinetics, a third dose was given to the animals. When the humanized version of 92R was used (Srb1), the animals received four doses of 16 mg/kg of either Srb1 or its isotype control (IgG1), unless otherwise indicated. The first dose was injected into the animals on the first week after injection of the tumor cells (days 2-7) and the following doses at weekly intervals afterwards. For some experiments the animals were treated intravenously with a single dose of vincristine (0.6 mg/Kg, Merck). On some experiments, mice were sacrificed simultaneously, the spleens and livers were photographed with a UV-light stereomicroscope. Subsequently, spleens and bone marrow were disaggregated, then cells were counted and FACS analyzed. For other experiments, the animals were maintained to determine the effects of 92R or Srb1 in survival. In these cases, the animals were sacrificed if they showed a significant decrease on body-weight or signs of diarrhea, behavior alterations, postural changes or locomotion deficiencies (end-point criteria).

For subcutaneous xenotransplants, HEK293T-CCR9GFP cells (2.5 x10^5^) were inoculated in the flank of NSG mice on day 0. The animals were divided into two groups, which were inoculated i.p. with the 92R or isotype control mAbs (4 mg/kg), the indicated days. The animals were then sacrificed and tumors were weighted and photographed. Similarly, subcutaneous xenotransplants were made in NSG mice with AsPC-1 cells (0.9x10^6^). The animals were divided into two treatment groups, which were inoculated with 3 intraperitoneal doses (4 mg/kg) of either 92R or isotype control mAbs at the indicated times. The animals were sacrificed on day 36 and tumors were dissected and weighted.

### 2.4 Analysis of tumor cells in liver cell suspensions

The liver cells were isolated as described (34). Briefly, animals were sacrificed, the livers extracted and homogenized using the GentleMACS™ dissociator using the program mLiver_01_03 at room temperature in 5 ml of PBS-glucose (4.5 g/L) supplemented with 100 µl of Collagenase A (Roche Diagnostics, 100 mg/ml) and 10 Units/ml of RQI-DNase I (Promega). The samples were digested for 30 min at 37°C followed with a second homogenization using the mLiver_02_03 program. Cells were washed, filtered through a 70 µm nylon mesh (Greiner). After lysis of the erythrocytes with isotonic ammonium chloride, the cell suspensions were FACS analyzed, gating on the live cell population.

On experiments using MOLT-4-luc cells (35) carrying a luciferase expression vector, orthotopic xenotransplants were generated with 2x10^5^ cells/mice; the animals were treated on days 35 and 37 with either 92R or isotype control mAb (100 µg/dose) and sacrificed on day 41. Livers were extracted and homogenized in a dounce homogenizer with a loose pestle in 2 ml of 1x cell culture lysis reagent (25 mM Tris HCl pH 7.8 with H_3_PO_4_, 2 mM EDTA, 2 mM DTT, 10% glycerol, 1% Triton X-100) (Promega # E153A). The luciferase activity on aliquots of the lysate was determined, following the manufacturer’s protocol, using Luciferase Assay Reagent, composed of 270 µM Coenzyme A (lithium salt), 470 µM luciferin, 530 µM ATP, 20 mM Tricine, 1.07 mM (MgCO_3_)_4_ Mg(OH)_2_-5H_2_O, 2.67 mM MgSO_4_, 0.1 mM EDTA, 33.3 mM DTT and final pH is 7.8 (Promega # E148A) and quantified in a GloMax^®^-Multi Detection System (Promega).

### 2.5 Metadata analysis for CCR9 expression

The metadata was obtained from the GENT2 public database (gene expression of normal and tumoral tissues), which contains normalized expression profiling data from human primary tumors and normal tissue for more than 68000 samples and additional features including prognostic value of the gene of interest for a subset of these samples (36). The data on the database is expressed as a log_2_ transformation of the expression values. For some of the analyses, the values were transformed to expression values.

### 2.6 Statistical analyses

Statistical analyses were performed using SPSS26 and SPSS28 software (IBM). Survival analyses used the Kaplan-Meier survival test. Statistical significance of the treatments was determined using the Chi-Square test. Non-parametric analyses of total cellularity, tumor cell numbers in spleen and bone marrow and tumor size in subcutaneous xenotransplants were determined using the Mann-Whitney U test. Statistical significance was established at P ≤ 0.05.

## 3 Results

### 3.1 Significance of CCR9 expression in T-ALL and other cancers

**Figure 1:**
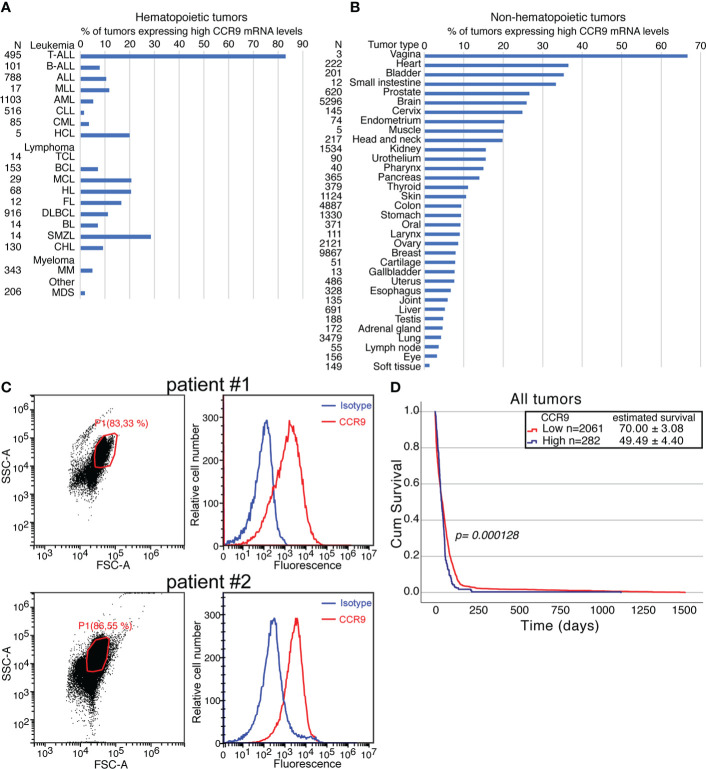
Significance of CCR9 expression in T-ALL and other cancers. CCR9 expression levels analyzed on tumor samples from the GENT2 database. For the analysis, the background for CCR9 mRNA was set up at 10. High expression levels were set up at levels higher or equal to 10-fold the background and low CCR9 expression at levels lower than 10-fold the background. **(A)** percentage of hematopoietic tumors expressing high CCR9 levels. The abbreviations used in this panel are: TALL, T cell acute lymphoblastic leukemia; BALL, B-cell acute lymphoblastic leukemia; ALL, Acute lymphoblastic leukemia; MLL, Mixed lineage leukemia; AML, Acute myeloid leukemia; CLL, Chronic lymphocytic leukemia; CML, Chronic myeloid leukemia; HCL, Hairy cell leukemia; TCL, T-cell Lymphoma; BCL, B-cell lymphoma; MCL, Mantle cell lymphoma; HL, Hodgkin lymphoma; FL, Follicular lymphoma; DLBCL, Diffuse large B-cell lymphoma; BL, Burkitt lymphoma; SMZL, Splenic marginal zone lymphoma; CHL, Chronic Hodgkin Lymphoma; MM, Multiple myeloma; MDS, Myelodysplastic syndrome. **(B)** percentage of non-hematopoietic tumors expressing high CCR9 levels. **(C)** Expression of CCR9 protein measured by flow cytometry in peripheral blood mononuclear cells (PBMC) from two patients diagnosed of T-ALL. Left panels FCS/SSC analyses of the samples, with the gates used for CCR9 analyses and the % of cells in the gate indicated. Right panels show the staining with an isotype control (blue) or 92R (red) revealed with an A647-anti-mouse IgG antibody **(D)** Effect of high or low expression CCR9 levels on survival determined on Kaplan-Meier survival curves. Statistical significance of the differences in the survival curves due to the treatment was determined using the Chi-Square test.

CCR9 expression levels were analyzed on normal and tumor samples of the GENT2 database. First the background value of expression was set on an expression of 10. Afterwards high expression values were defined as expression levels being ≥ 10-fold the background and low expression values as expression levels being < 10-fold the background. Using this cut-off, we could determine that from 495 tumor samples of T-ALL patients present in GENT2, >80% of them expressed high levels of CCR9 mRNA. The expression mRNA levels for CCR9 in other leukemias, lymphomas or myelomas is much lower, although about 30% of the tumor samples in Splenic marginal zone lymphoma and about 20% of the samples in Hairy cell leukemia, Mantle cell lymphoma or Hodgkin lymphoma express high levels of CCR9 mRNA (see **Figure 1A**). It should be noted that the CCR9 expression levels on hematopoietic and non-hematopoietic tumor samples have the same order of magnitude (1-8000 for hematopoietic and 1-2000 for non-hematopoietic tumor samples). On non-hematopoietic tissues, high levels of CCR9 mRNA expression are found on >20% of the samples from heart, bladder, small intestine, prostate, brain, cervix, endometrium, muscle and head and neck tumors, whereas on kidney, urothelium, pharynx, pancreas, thyroid and skin tumors CCR9 mRNA is over-expressed in >10% of the analyzed tumor samples (**Figure 1B**). The high levels of CCR9 mRNA expression in T-ALL correlate well with the expression levels detected by flow cytometry on peripheral blood mononuclear cells obtained from blood samples of two patients diagnosed of T-ALL (**Figure 1C**). Furthermore, since there were no data available on survival for T-ALL samples on GENT2, we decided to analyze the survival of all tumor patients with available data, depending on CCR9 expression levels. The data is from 2061 samples for low CCR9 expression and 282 for high CCR9 mRNA expression. The survival data show significant differences between low and high CCR9 expressing tumors *(p=0.000128)*, with estimated survivals of 70.0 ± 3.1 and 49.5 ± 4.4 days, respectively (**Figure 1D**). Furthermore, an analysis of the expression of CCR9 in a panel of tumor cell lines shows that some of them do not express CCR9, whereas others, including the colorectal carcinoma (KM12L4), the melanoma A375, the glioblastoma U87, the ovarian adenocarcinoma SKOV3, the lung carcinoma A549, the mammary adenocarcinoma MCF7 and the pancreas carcinoma AsPC-1 express CCR9 on their surface (**Supplementary Figure 1**). Taken together, these data indicate the relevance of CCR9 expression levels in hematopoietic and non-hematopoietic tumors, as well as its effects on survival, suggesting that CCR9 might represent a relevant therapeutic target. The expression of CCR9 at the mRNA and protein levels in T-ALL has been recently confirmed by others (37).

### 3.2 Effect of 92R mAb on tumor burden and survival

The anti-human CCR9 mAb 92R, which recognizes the human CCR9, but not the mouse CCR9, inhibits the growth of subcutaneous xenografts generated with the human T-ALL cell line MOLT-4 (35). To analyze the robustness of a treatment with 92R mAb, it is necessary to demonstrate whether this mAb is able to inhibit the growth of T-ALL orthotopic tumor xenografts.

**Figure 2:**
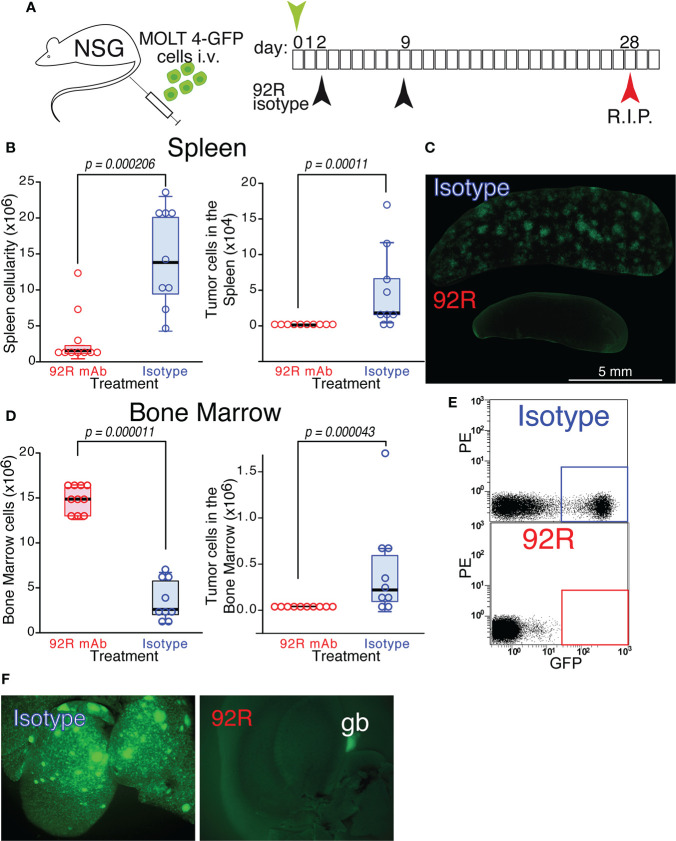
Effect of 92R mAb on the progression of MOLT4-GFP orthotopic xenotransplants. **(A)** Schematic representation of the experimental set-up. MOLT4-GFP cells (1×10^6^) were injected in the tail vein of NSG mice. The mice were separated into two groups that were treated on days 2 and 9 with either 92R mAb (100 µg/dose, n=10) or with an isotype control mAb (mouse IgG2a), (100 µg/dose, n=9). All the animals were sacrificed on day 28. **(B)** Total cellularity and tumor cell number in the spleen. **(C)** Stereomicroscopic UV images of a representative spleen from each treatment group, where the accumulation of tumor cells (GFP^+^) in the isotype control treated animals, but not in the 92R mAb-treated group, could be readily observed. **(D)** Total cellularity and tumor cell number in the bone marrow (BM) of xenotransplanted animals treated with either 92R or isotype control mAb. **(E)** Representative flow cytometry analyses showing the tumor cells in the BM (quantified as the fraction of GFP^+^ cells) on isotype control and 92R mAb-treated animals. **(F)** UV-stereomicroscopic images of representative livers from NSG animals carrying MOLT4-GFP xenotransplants treated with either control isotype mAb or 92R mAb. The gallbladder (gb) visible in the 92R-treated liver is due to autofluorescence. **(B, D)** One-tailed Mann-Whitney U test. Statistical significance was established in p <0.05.

CCR9^+^ MOLT4-GFP cells were injected on NSG mice to generate orthotopic xenotransplants. The mice were divided into two groups that were treated on days 2 and 9 with either 92R mAb or with an isotype control mAb and sacrificed on day 28 (**Figure 2A**). The median of the spleen total cellularity was 7.9 times higher in the isotype control than in the 92R treated mice (p=0.000206) (**Figure 2B**, **Table 1**). The tumor cell number in the spleen was significantly higher in the isotype control than in 92R treated group (p=0.00011) (**Figure 2B**, **Table 1**). These results were corroborated with UV-light stereomicroscopic images of the spleens, where the accumulation of tumor cells is visible in the isotype control treated animals but remained undetected on the 92R treated group (**Figure 2C**). In the bone marrow, the two femurs for each animal were always analyzed. The results indicate that the situation is somehow different than in the spleen, since in 92R mAb treated animals, the total cellularity was 5.7-fold higher than in the isotype control treated group (p=0.000011), although the tumor cell number was lower (**Figure 2D**), as shown on a representative flow cytometry analysis of the bone marrow of animals treated with isotype control mAb or with 92R, where the human tumor cells could be distinguished from the endogenous mouse bone marrow cells by GFP expression (**Figure 2E**). In addition, an accumulation of MOLT4-GFP cells was observed in the livers of isotype control treated animals, but could not be detected in the livers of 92R mAb treated mice (**Figure 2F** and **Supplementary Figure 2A**). Interestingly, the tumor cell infiltrates in the livers of MOLT4-GFP xenografts displayed a completely different pattern than the infiltrates in the livers of xenotransplants from the T-cell leukemia cell line Jurkat-GFP (selected for being CCR9^-^), as demonstrated by UV-stereomicroscopic imaging (**Supplementary Figures 2A, B**). Furthermore, the MOLT4-GFP-tumor cell infiltrates in the livers of the xenotransplanted animals was abrogated by 92R treatment (**Figure 2F** and **Supplementary Figure 2A**), whereas the liver infiltrates of the CCR9^-^ Jurkat-GFP tumor cells in the corresponding xenotransplants displayed a completely different pattern and remained unchanged when comparing isotype control and 92R mAb treated animals (**Supplementary Figure 2B**).

**Table 1:** Total and tumor cell analyses in spleen and bone marrow of orthotopic xenotransplanted mice treated with 92R.

	isotype control mAb	92R mAb	p- value**	medianfold-change
Spleen				
Total Cellularity* (median) (x10^6^) range (x10^6^) [min - max] (x10^6^)	14.2 19.04 [4.5 - 23.6]	1.8 11.7 [12 - 31.6]	0.000206	7.9
Tumor cells (median) (x10^6^)	1.7 x10^4^	0	0.000011	n.d.***
range (x10^6^) [min - max] (x10^6^)	0.166 [0.003 – 0.17]	2468 [0-0.002]		
Individuals/group (n)	9	10	–	–
Bone Marrow				
Total cell number (median) (x10^6^) range (x10^6^) [min – max] (x10^6^)	2.63 5.8 [1 – 6.8]	15.1 3.9 [13 – 17]	0.000011	0.17
Tumor cells (median) (x10^6^)	0.246	0	0.000043	n.d.***
range (x10^6^) [min - max] (x10^6^)	1.7 [0.002-1.7]	0.0046 [0 – 0.0046]		
Individuals/group (n)	9	10	–	–

*the distribution of the values is not normal, therefore the values used for comparison are the median and where appropriate the range of values.

**The statistics analyses were done using the Mann-Whitney U test (one tail) and the exact p value was calculated.

***n.d. not determined since one of the values was 0.

**Figure 3:**
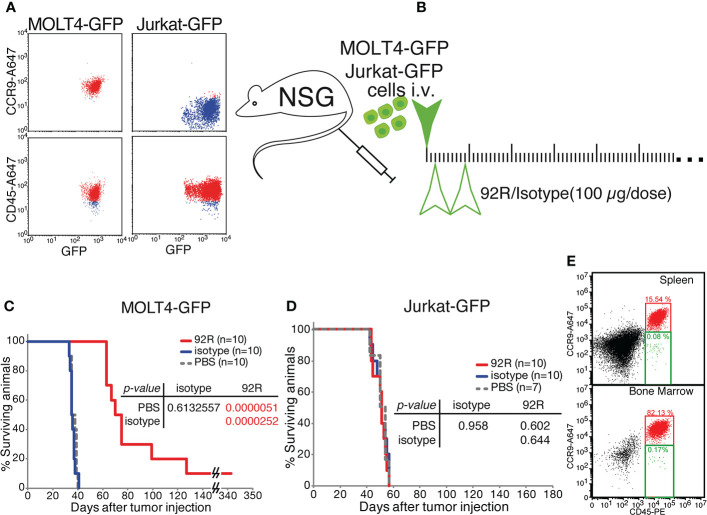
Effects of 92R mAb on survival. Two different T-ALL cell lines were used for the survival analyses, the MOLT4-GPF cells were used as a model of CCR9^+^ T-ALL and the Jurkat-GFP cells as a model of CCR9^-^ T-ALL. **(A)** Flow cytometric analyses of MOLT4-GFP and Jurkat-GFP to verify the expression of CD45 (human cell marker) and GFP, and to demonstrate the expression of CCR9 on the MOLT4-GFP, and the lack of expression of the receptor on Jurkat-GFP. **(B)** Schematic representation of the survival experiments where the tumor cells, either MOLT4-GFP or Jurkat-GFP (1×10^6^ cells) were injected in the tail vein of NSG mice on day 0, The mice were separated into three different groups and inoculated intraperitoneally on days 2 and 9 with either 92R mAb (4 mg/kg; red), isotype control mAb (4 mg/kg; blue) or PBS (100 µl; gray dashed line). Statistical significance of the differences in the survival curves due to the treatment was determined using the Chi-Square test **(C)**. Kaplan-Meier survival curves from the experiment using MOLT4-GFP cells (n=10 for each treatment). **(D)**. Kaplan-Meier survival curves from the experiment using Jurkat-GFP cells (n=10 for 92R; n=10 for Isotype; n=7 for PBS). **(E)** Flow cytometry analyses of the spleen and bone marrow from a representative mouse with a MOLT4-GFP xenotransplant, treated with 92R and maintained until weight loss was initially observed (48-72 hours before death). The single cell suspensions were simultaneously stained with anti-CD45-PE and biotinylated anti-CCR9 mAb that was revealed with streptavidin-Alexa647.

We next investigated if the decrease in tumor cell numbers observed on the MOLT4-GFP xenotransplanted animals treated with 92R is also reflected in survival differences. For this purpose, two different T-ALL cell lines were used, the MOLT4-GPF cells as a model of CCR9^+^ T-ALL and the Jurkat-GFP cells as a model of CCR9^-^ T-ALL. Expression of human CD45 and GFP by both cell lines and differential expression of CCR9 was demonstrated by flow cytometry analyses (**Figure 3A**). For the survival experiments (**Figure 3B**), either MOLT4-GFP or Jurkat-GFP tumor were injected in the tail vein of NSG mice that were separated into three different treatment groups: 92R, isotype control and PBS Analyses of the Kaplan-Meier survival curves from the experiment using MOLT4-GFP cells indicate that the groups of mice treated with either isotype control or PBS have estimated survival times of 36.70 ± 0.80 and 36.10 ± 0.72 days, respectively, whereas 92R mAb treated animals have an estimated survival of 92.50 ± 15.05 days (p= 0.0000051, and p= 0.0000252, respectively), demonstrating a 2.6-fold survival increase on the 92R-treated group (**Figure 3C**). The specificity of 92R mAb treatment was demonstrated with mice carrying xenografts generated with the CCR9^-^Jurkat-GFP cells and the same treatments (92R, isotype control or PBS). The Kaplan-Meier survival curves fail to exhibit significant survival differences (estimated mean survivals of 52.10 ± 1.62, 52.90 ± 1.62 and 53.57 ± 1.85 days, respectively) (**Figure 3D**). Thus, showing the specificity of 92R effects on CCR9 expressing tumors, and indicating that 92R and the isotype control mAbs, at the concentrations used in the assay conditions, are devoid of toxicity on mice (**Figure 3D**). Furthermore, we found that after treatment with 92R mAb, the tumor cells present in the spleen and the liver did not have the CCR9 expression altered by the treatment. Indeed, in the spleen cell suspension, 84.38% of the cells represent endogenous mouse cells and the remaining 15.62% of the cells represent tumor cells. From the tumor cells, the 99.4% were CD45^+^CCR9^+^ and only the 0.5% of the tumor cells were CD45^+^ with a decreased expression of CCR9. For the bone marrow, 17.7% of the cells represent endogenous mouse cells and 82.3% represent tumor cells, where 99.8% were CD45^+^CCR9^+^ and only the 0.02% of the tumor cells were CD45^+^ with a decreased expression of CCR9 (**Figure 3E**). Thus, the expression levels of CCR9 did not seem altered after treatment of the mice with 92R mAb and suggests that different strategies of administration or higher doses could be more efficient to reduce tumor burden.

The animal censored on day 342 in **Figure 3C**, was sacrificed and spleen cell samples were analyzed by flow cytometry, where only one tumor cell was detected out of 50 000 cells analyzed, whereas we failed to detect any tumor cell in the bone marrow (<1/50 000) (**Supplementary Figures 2C, D**). Stereomicroscopic UV-images of the spleen did not show any accumulation of tumor cells (**Supplementary Figure 2E**) confirming the flow cytometry data.

**Figure 4:**
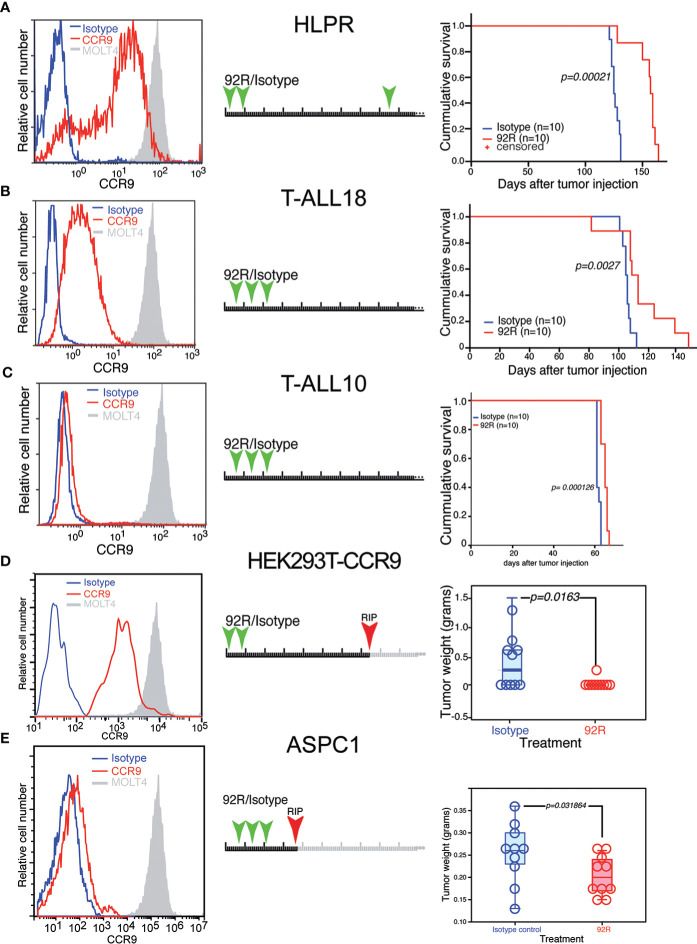
Effect of 92R on the progression of primary human T-cell leukemias and on non-hematopoietic CCR9-expressing solid tumors. **(A–C)** Three primary T-ALL leukemias were analyzed for CCR9 expression (left panels), HLPR expressed 3.6-fold less CCR9 **(A)**, T-ALL18 expressed 17.5-fold less **(B)** and T-ALL10 expressed >50 fold less CCR9 than MOLT4 cells **(C)**. For survival experiments, 1x10^6^ primary tumor cells of HLPR (n=10 mice per group) **(A)**, T-ALL18 (n=9 mice per group) **(B)** or T-ALL 10 (n=10 mice per group) were injected in the tail vein of NSG mice. The animals were divided into two treatment groups and treated with 4 mg/kg/dose of 92R mAb (red) or isotype control mAb (blue) on days 2, 9 and 85 **(A)**, on days 4, 11 and 18 **(B, C)** as described in the schematic representation of each experiment. The animals treated with 92R on each xenotransplant experiment had a significant increase on survival as compared to the corresponding control group. Data on **(A–C)** were analyzed using the Kaplan-Meier survival test. Significance of the differences in the survival curves due to the treatment was determined using the Chi-Square test. Effect of 92R in non-hematopoietic CCR9^+^ tumors. **(D)** Stable expression of a human CCR9-GFP fusion protein was forced through transfection into the human embryonic kidney HEK293T cell line. Stable transfectants were FACS sorted by CCR9 expression to avoid using cells expressing high levels of the fusion protein in an intracellular compartment, and the cells were subcutaneously injected into the flanks of NSG mice. The animals were treated on days 1 and 8 with either 92R or isotype control mAbs. After development of the tumors, animals were sacrificed on day 74. The weight of the tumors from IgG2a-isotype control (n=10) or 92R-treated mice (n=10) at the time of sacrifice were analyzed using the two-tailed Mann-Whitney U test **(D)**. **(E)** expression levels of CCR9 on the surface of the pancreatic carcinoma cell line AsPC-1. These cells were used to generate subcutaneous xenotransplants in NSG mice. The animals were treated on days 7, 14 and 21 with either 92R (n=10) or isotype control (n=10) mAbs and were sacrificed on day 36. After sacrifice, the tumors were dissected and weighted. The data was analyzed using the two-tailed Mann-Whitney U test **(E)**. Statistical significance was established in p <0.05.

We next investigated the therapeutic effect of 92R mAb on primary human T-cell leukemia (HLPR, T-ALL18 and T-ALL10) expressing different CCR9 levels. Indeed, animals transplanted with the T-cell precursor lymphoblastic leukemia HLPR, which displays 3.6-fold lower CCR9 expression levels than MOLT4 cells in 85% of the cells (**Figure 4A**), showed a significant survival increase (p=0.00021) upon treatment with 92R mAb as compared to the isotype control-treated group (estimated survivals of 154 ± 4.02 and 126 ± 1.1353 days, respectively) (**Figure 4A**). In xenotransplantation assays with the primary T-ALL18, expressing 17.5-fold lower CCR9 levels than MOLT4 cells (**Figure 4B**), treatment with 92R still showed significant (p=0.0027) survival differences with the isotype control mAb treated group (estimated survivals of 105.3 ± 1.2 and 115.9 ± 6.3 days, respectively) (**Figure 4B**). Finally, xenotransplants with a third primary T-ALL (T-ALL10) expressing ~1% of the CCR9 levels present in the MOLT4 cells (**Figure 4C**), treatment with 92R mAb still showed significant (p=0.000126) survival differences with the isotype control mAb treated group (estimated survivals of 61.7 ± 0.3 and 64.9 ± 0.5 days, respectively) (**Figure 4C**) Taken together, these data suggest that the treatment with 92R mAb is also effective in primary T-ALL cells, and the increase on survival directly correlates with the expression levels of CCR9 on the surface of the cells.

Since CCR9 can also be over-expressed in some non-hematopoietic tumors, the effect of 92R mAb on tumor burden was analyzed in HEK293T cells over-expressing a CCR9-GFP construct. Subcutaneous xenotransplants in NSG mice were generated after sorting the CCR9^+^GFP^+^ HEK293Tcells, the animals were treated with either 92R mAb or isotype control mAb a and sacrificed on day 74 (**Figure 4D**). Five out of 10 mice from the isotype control treated group bear subcutaneous tumors and only 1 out of 10 mice from the 92R mAb treated group carry a tumor. Quantification of the tumor weights demonstrated significant differences (p=0.0163; Mann-Whitney U test) (**Figure 4D**). Furthermore, since the screening of tumor cell lines identified the pancreas adenocarcinoma tumor cell line AsPC-1 as expressing CCR9 on its surface, we conducted an *in vivo* experiment using subcutaneous xenotransplants of these cells into NSG mice. Ten animals were treated with three doses of either 92R or isotype control antibody, and all of them sacrificed on day 36. The subcutaneous tumors were dissected and weighted. Quantification of the tumor weights demonstrated significant differences (p=0.031864; Mann-Whitney U test), with the animals from the 92R-treatment group having lower weights (**Figure 4E**). Taken together, these data show that 92R mAb could also impair progression of non-hematopoietic CCR9^+^ tumors, in particular of pancreatic tumors.

### 3.3 Effect of a humanized version of 92R (Srb1) on the treatment of MOLT4-GFP xenotransplants

**Figure 5:**
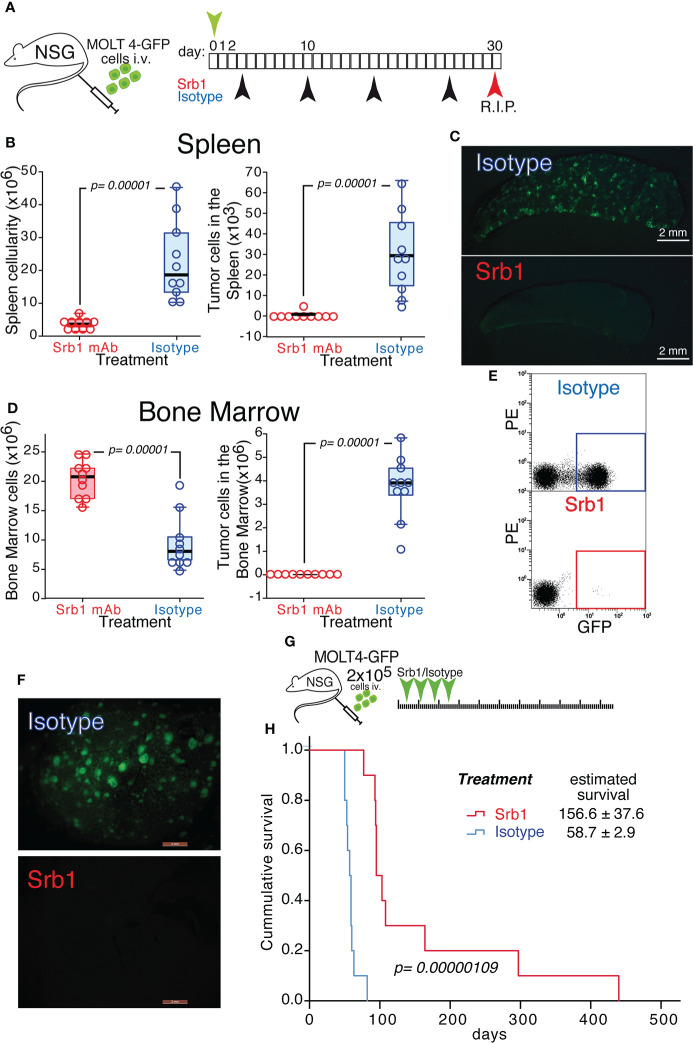
Effect of Srb1 mAb on the progression of MOLT4-GFP orthotopic xenotransplants. **(A)** Schematic representation of the experimental set-up. MOLT4-GFP cells (1×10^6^) were injected in the tail vein of NSG mice. The mice were separated into two treatment groups that were treated on days 3, 10, 17 and 24 with Srb1 mAb (16 mg/kg; n=10) or with an isotype control mAb (16 mg/kg; n=10). All the animals were sacrificed on day 30. **(B)** Total cellularity and tumor cell number in the spleen. **(C)** Stereomicroscopic UV image of a representative spleen from the isotype control treated group, where the accumulation of tumor cells (GFP^+^) could be readily observed. **(D)** Total cellularity and tumor cell number in the bone marrow (BM). **(E)** Representative flow cytometry analyses showing the tumor cells in the BM (quantified as the fraction of GFP^+^ cells) on isotype control and Srb1 mAb-treated animals (further information is shown in **Supplementary Figure 3A**). **(F)** UV-stereomicroscopic representative liver images from an animal treated with isotype control mAb (top) and an animal treated with Srb1 (further information is shown in **Supplementary Figure 3B**). **(G)** Schematic representation of the experiment designed to determine survival in response to Srb1 treatment. 2×10^5^ MOLT4-GFP cells were injected in the tail vein of NSG animals on day 0. The mice were separated into two different groups and treated on days 4, 11, 18 and 25 intraperitoneally with either Srb1 mAb (16 mg/kg; n=10; red), or isotype control mAb (16 mg/kg; n=10; blue). **(H)** Kaplan-Meier survival curves of the animals from this experiment, indicating the mean survival ± SD, as well as the statistical significance of the differences in the survival curves, determined using the Chi-Square test. **(B, D)** One-tailed Mann-Whitney U test. Statistical significance was established in p <0.05.

We next investigated whether the effects on orthotopic xenotransplants described for 92R mAb could also be observed using a humanized version of this antibody (Srb1). For this experiment, MOLT4-GFP cells were intravenously injected into NSG mice. The animals were divided into two treatment groups Srb1 and isotype-control and sacrificed on day 30 after tumor injection (**Figure 5A**). In the spleen of isotype control-treated mice, the median cellularity was 5.22-fold higher than in the Srb1-treated mice (p=0.00001), whereas the tumor cell number was 37.35-fold higher (p=0.00001)(**Figure 5B**, **Table 2**). The data on tumor cellularity were corroborated by UV-light-stereomicroscopic images of the spleens from these animals (**Figure 5C**). Analysis of the bone marrow, showed a 2.56-times higher median cellularity in the Srb1-treated group, with a median of tumor cells 1,761-fold lower than in isotype control treated animals (p=0.00001) (**Figure 5D**, **Table 2**). In addition, **Figure 5E** shows a representative flow cytometry analysis of the bone marrow cells, which demonstrate the differences in tumor cell numbers shown in **Figure 5D**. Indeed, in the isotype control treated group, the bone marrow contained up to 60% of tumor cells, whereas they were practically undetectable in the Srb1-treated group (**Supplementary Figure 3A**).

**Table 2:** Total and tumor cell analyses in spleen and bone marrow of orthotopic xenotransplanted mice treated with Srb1.

	h-isotype control Ab	Srb1 Ab	p- value**	fold-change
Spleen				
Total Cellularity* (median) (x10^6^) Range (x10^6^) [min – max] (x10^6^)	18.7 35.2 [10.3 – 45.5]	3.6 5.6 [1.4 – 6.9]	0.00001	5.22
Tumor cells (median) (x10^6^) range (x10^6^) [min – max] (x10^6^)	0.029 0.059 [0.007 – 0.066]	0.0008 0.0047 [0 – 0.0047]	0.00001	37.35
Individuals/group (n)	10	10	–	–
Bone Marrow				
Total cell number (median) (x10^6^) range (x10^6^) [min – max] (x10^6^)	8.1 14.4 [4.9 – 19.3]	20.8 9.0 [15.6 – 24.6]	0.00004	0.39
Tumor cells (median) (x10^6^) range (x10^6^) [min – max] (x10^6^)	3.9 x10^6^ 4.8 [1.1 – 5.8]	2220 0.03 [0 – 0.03]	0.00001	1760.9
Individuals/group (n)	10	10	–	–

*The distribution of the values is not normal, therefore the values used for comparison are the medians.

**The statistics analyses were done using the Mann-Whitney U test (one tail) and the exact p value was calculated.

In addition, the livers from all the animals in the isotype control treated group showed infiltration of MOLT4-GFP cells, whereas the Srb1-treated group did not show tumor cell infiltration in their livers (**Figure 5F** and **Supplementary Figure 3B**). Thus, both 92R mAb and Srb1 show their effectiveness inhibiting tumor cell infiltrations in the livers of xenotransplanted animals. Furthermore, the decrease of tumor cells in the group treated with Srb1 correlated with a 2.66-fold survival increase (p=0.00000692), as compared with the isotype control treated animals (from 156.6 ± 37.6 days to 58.7 ± 2.9 days, respectively) (**Figures 5G, H**). Taken together, these data indicate that Srb1, under the conditions used, is as effective as 92R decreasing tumor cellularity in bone marrow and spleen, tumor cell infiltration in the liver and increasing survival of the xenotransplanted animals.

### 3.4 Effect of 92R and Srb1 on MOLT4-GFP liver infiltrates of xenotransplanted animals

**Figure 6:**
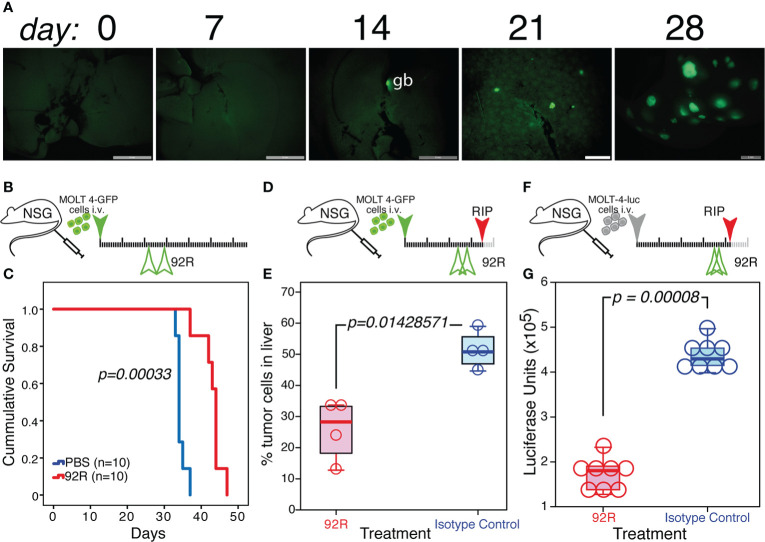
Effect of 92R mAb on liver infiltrates induced by MOLT4-GFP orthotopic xenotransplants. **(A)** UV-stereomicroscopic images of mouse livers at days 7, 14, 21 and 28 after intravenous injection of MOLT4-GFP cells (1×10^6^), where liver infiltrates of tumor cells were detectable on day 21. **(B, C)** Effect of a delayed 92R-treatment started after the appearance of tumor cells in liver infiltrates. **(B)** Schematic representation of the experimental set-up. The MOLT4-GFP cells (1×10^6^) were injected in the tail vein of NSG mice. The mice were treated on days 22 and 29 with 92R mAb (4 mg/kg; n=10) or with PBS (n=10). **(C)** Kaplan-Meier survival curves of these animals. Statistical significance of the differences in the survival curves due to the treatment was determined using the Chi-Square test. **(D, E)** Effects of the treatment on the infiltrating tumor cells in the liver. **(D)** Schematic representation of the experimental set-up. The mice were treated on days 24 and 29 with 92R mAb (4 mg/kg; n=4) or with the same dose of isotype control mAb (n=4) and sacrificed on day 35. **(E)** Livers were disaggregated and the fraction of tumor cells present in them was analyzed by flow cytometry, gating on living cells and detecting the tumor cells by their expression of the GFP marker. **(F, G)** Effects of 92R treatment on MOLT-4-luc cells in the liver. **(F)** Schematic representation of the experimental set-up. The mice were injected on the tail vein with 2x10^5^ MOLT-4-luc cells expressing luciferase on day 0. Afterwards, they were treated on days 35 and 39 with either 92R mAb (4 mg/kg; n=8) or the same dose of isotype control mAb (n=8) and sacrificed on day 42. **(G)** Livers were extracted and homogenized in luciferase lysis buffer and the luciferase activity quantified on equal liver aliquots. The data from E and G were analyzed using a Two-tailed Mann-Whitney U test. Statistical significance was established in p <0.05. gb indicates the gallbladder, which in all cases is auto-fluorescent.

Whereas the Jurkat-GFP tumor cell infiltrates in the liver do not change upon 92R mAb-treatment (**Supplementary Figure 2B**), the infiltrates of MOLT4-GFP cells in the liver of isotype control treated animals shows a completely different distribution pattern and is abrogated by treatment with either 92R mAb or Srb1 (**Supplementary Figures 2A, 3B**). Aiming to investigate whether the liver infiltrations of MOLT4-GFP cells in xenotransplanted mice corresponded to a primary organ colonization, or rather the result of a secondary colonization, the kinetics of liver colonization was analyzed. The obtained results show that infiltrates of tumor cells are not detected in the liver before day 21 (**Figure 6A**), a result that suggests that the tumor cells initially colonize the spleen and bone marrow, and use the liver as a secondary settlement. The data presented so far, do not allow to distinguish whether the effects of 92R treatment are restricted to the inhibition of liver infiltrate formation, or conversely it can also reduce the number of tumor cells in already formed infiltrates. For this purpose, an experiment was carried out where mAb treatment started on day 22 (**Figure 6B**), this experiment shows that 92R mAb is still able to increase the survival of the MOLT4-GFP xenotransplanted animals by 30% (estimated survivals of 34.43 ± 0.48 days and 43.00 ± 1.16 days for PBS-treated and 92R-treated animals, respectively, p=0.00033) (**Figure 6C**). Furthermore, the effect of 92R mAb on the number of infiltrating tumor cells in the liver was analyzed in animals carrying MOLT4-GFP xenotransplants starting the treatment on day 25 with either 92R or isotype control mAbs (**Figure 6D**). On day 35, animals were sacrificed, the livers were extracted, digested to obtain a single cell suspension and the fraction of GFP^+^ leukemic cells in the samples analyzed by flow cytometry. The percentage of tumor cells in the livers of the animals treated with isotype control was significantly higher than on 92R mAb-treated animals (51.28% ± 6.01 compared to 25.75% ± 9.76, respectively, p=0.0143), indicating that 92R mAb treatment after the infiltrates have been generated, is still able to reduce the number of tumor cells within these liver infiltrates (**Figure 6E**). These data were confirmed on a complementary experiment where MOLT4 cells carrying a luciferase expression vector (MOLT-4-luc) were injected intravenously and the treatment started on day 35 with either 92R mAb or isotype control mAb and sacrificed on day 41 (**Figure 6F**). The livers from these animals were extracted and after homogenization in luciferase lysis buffer, aliquots were quantified for luciferase activity, as a direct estimate of the amount of infiltrating MOLT-4-luc cells in the livers. The results obtained show that the fraction of tumor cells present in the livers of the animals treated with isotype control was significantly higher than on 92R mAb-treated animals (p=0.00008) (**Figure 6G**).

### 3.5 Effect of Srb1 in combination with chemotherapy on survival of the xenotransplanted animals

**Figure 7:**
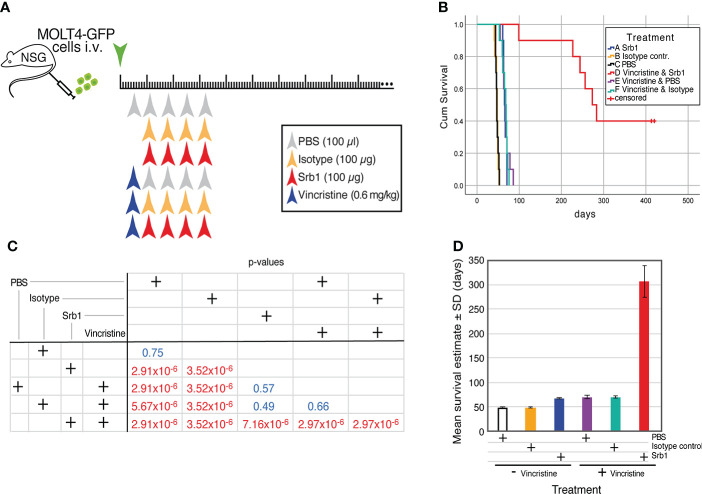
Effect of Srb1 in combination with vincristine on the survival of MOLT4-GFP orthotopic xenotransplants. **(A)** Schematic representation of the survival experiment where animals were treated with PBS, isotype control antibody, or Srb1, either in the absence or presence of the chemotherapeutic vincristine. 1×10^6^ MOLT4-GFP cells were injected in the tail vein of NSG animals on day 0, The mice were separated into six different treatment groups. Vincristine treatment (0.6 mg/kg) on a single i.v. dose on day 5; isotype control and Srb1 treatment (100 µg/dose, 4 mg/kg) on days 11, 18, 25 and 32, and PBS (100 µl) on day 5 as control for vincristine, and on days 11, 18, 25 and 32 as controls for Srb1. **(B)** Kaplan-Meier survival curves of the animals from this experiment, on the group combining vincristine and Srb1 there were 4 animals censored. **(C)** Chi-square test to determine the statistical significance of the survival differences between groups, where statistical significance was established at p <0.05. **(D)** Estimated mean survival ± SD from each group.

To determine the effects of Srb1 in combination with chemotherapeutic agents, MOLT4-GFP xenotransplanted animals were subdivided into 6 different groups, that were treated with PBS (100 µl), isotype control Ab (100 µg/dose) or Srb1 (100 µg/dose), either in the absence, or in the presence of the chemotherapeutic agent vincristine (0.6 mg/kg). The schematic representation of the experiment and treatment schedule is shown in **Figure 7A**. As shown in the Kaplan-Meier survival curves, despite the suboptimal concentration of Srb1 used, there is a significant survival increase (66.2 ± 1.17 days, as compared to 47.1 ± 1.09 and 47.6 ± 1.00 days, for Srb1, PBS or isotype control Ab treated groups, respectively). Similarly, a significant survival increase was observed upon vincristine treatment (67.9 ± 2.85), as compared to PBS treated animals. As expected, the survival effect of vincristine remained unchanged if it was used either alone or in combination with isotype control mAb (66.6 ± 2.19 days). However, a strong synergism could be observed when vincristine and Srb1 treatments were combined, since the mean survival of these animals increase to 306.2 ± 33.02 days (**Figure 7B**). The significance of the differences was determined by Chi-square analyses (**Figure 7C**), whereas **Figure 7D** shows the mean ± SD of survival upon each treatment.

## 4 Discussion

The expression of the CCR9 chemokine receptor gene is limited in normal tissues, but the analysis presented here demonstrates that CCR9 is over-expressed in >80% of T-ALL leukemia patients, and in a considerable fraction of other hematopoietic and non-hematopoietic tumors. Furthermore, the analysis of survival data on a limited amount of cancer patients showed a negative correlation between CCR9 expression levels and survival. Taken together, these data suggest that CCR9 might represent a relevant therapeutic target. T-ALL is a leukemia which, despite advances in treatment, still has a high percentage of patients showing resistance to chemotherapy and/or relapse (38). In these patients, the presence of CCR9 receptor in tumor cells has been associated with increased tissue infiltration and metastasis (2–4). We generated antibodies against CCR9, one of them, 92R mAb, was selected for its effectiveness inhibiting the growth of CCR9^+^ MOLT-4 subcutaneous xenografts as demonstrated in both Rag2^-/-^ and NSG immunodeficient mice (32). The robustness of 92R mAb treatment *in vivo*, was ascertained using orthotopic xenografts in NSG mice, since this model closely resembles human leukemia. Indeed, in NSG animals intravenously injected with MOLT4-GFP^+^ cells, the tumor cells could be detected circulating in the blood and accumulate both, in the spleen and bone marrow of these animals, as determined by flow cytometry analyses based on GFP expression of the tumor cells. Conversely, it could also be determined after staining the cells with an anti-hCD45 mAb. These experiments demonstrate that animals carrying MOLT4 orthotopic xenotransplants, when treated with only 2 doses of 4 mg/kg of 92R mAb show a drastic decrease in tumor cell numbers, both in the spleen and bone marrow. The decrease in the tumor cell number in 92R-treated mice is also reflected on a survival increase (2.6-fold) of the animals, as compared to isotype control mAb treated ones. The specificity of 92R treatment was demonstrated by the lack of effect on survival in mice xenotransplanted with a CCR9^-^ T-ALL cell line (Jurkat).

Similar results were obtained when the MOLT4-GFP xenotransplants were treated with Srb1, a humanized version of 92R mAb. In this case, the mice were treated with 4 doses of antibody of 16 mg/kg, due to the reduced half-life of human IgG in mice. Under these conditions, the Srb1 treated group had lower tumor cell numbers in spleen and bone marrow (1,760-fold lower) than the isotype control-treated animals. The decrease in tumor cells also correlate with a 2.66-fold increase in animal survival in the Srb1-treated group.

The increased survival *in vivo* of 92R mAb-treated animals, rather than restricted to xenografts of the MOLT-4 cell line, was also observed in orthotopic xenografts generated with primary CCR9^+^ T-ALL leukemias, as demonstrated with HLPR, T-ALL18, and T-ALL10, despite the lower expression levels of CCR9 on the surface of these primary leukemia cells (3.6-fold,17.5-fold and >50-fold lower than in the MOLT-4 cells). Furthermore, and despite the significance of the differences between 92R and isotype control treated animal groups, it is worth to note that the magnitude of the survival effect correlated positively with the CCR9 expression levels. In addition, on non-hematopoietic tumors with forced CCR9 expression, 92R treatment had also an effect decreasing the number of subcutaneous tumor-carrying animals, as well as the tumor’s size, as demonstrated with HEK293T-CCR9 derived solid tumors as a proof-of-principle. In addition, in subcutaneous xenotransplants using the pancreas carcinoma cell line AsPC-1 (33), which expresses CCR9 on its surface, there was a significant difference on the weight of the tumors. These data are highly relevant, in light of the relevance of pancreatic cancer since it represents 2.9% of the newly diagnosed tumors/year with about 500,000 newly diagnosed tumors/year, and its mortality-to-incidence ratio (MIR) which is 94%, the highest for any type of tumor (*GLOBOCAN2020 data, presented by the International Agency for Research on Cancer* at https://gco.iarc.fr/today/home*).* Furthermore, analyses of the data from the public GENT (Gene Expression across Normal and Tumor Tissue) database (39) showed that CCR9 mRNA is over-expressed in 19.8% hematological tumors (11,001 analyzed), and in 11.3% non-hematological tumors (16 258 analyzed) (M. Delgado, M. Botas, S. Santamaria, L. Kremer, P. Garrido & J.A. Garcia-Sanz, in preparation). It should be noted that further optimization of mAb doses and administration frequencies might boost the observed differences in both hematopoietic and non-hematopoietic tumors between 92R mAb treated and isotype control mAb treated groups.

In addition, unlike in the spleen, the total bone marrow cellularity of 92R- and Srb1-treated animals is significantly higher than in the isotype control-treated animals. The increased bone marrow cellularity was reproducibly observed in different experiments, despite the lower tumor cell numbers. The higher cellularity in the bone marrow of 92R- and Srb1-treated animals is due to endogenous cells and might simply be the consequence of a decrease in bone marrow cellularity on the isotype control treated animals, since the tumor cells, with a 4 to 5-fold larger volume than the endogenous bone marrow cells, have successfully replaced a large fraction of the endogenous bone marrow precursors, while competing for a restricted space, reducing the ability of the bone marrow to produce red blood cells and platelets, as it has been described to occur in humans (40). Conversely, it is also possible that these endogenous cells in the bone marrow of 92R- and Srb1-treated animals might represent activated phagocytic cells involved in tumor cell depletion, as it has been previously proposed (32). Further analyses are required to distinguish between these possibilities.

The data presented here also show that, in MOLT-4 generated xenografts, there is an accumulation of tumor cells in the liver, representing liver infiltrates. Hepatic involvement in acute leukemia has been described as usually mild and silent at diagnosis, but liver infiltration has been described in over 95% of acute lymphoblastic leukemia (ALL) cases postmortem (41). In the MOLT-4 xenotransplants the tumor cell liver infiltrates had a distinct pattern from the infiltrations observed in Jurkat-derived xenografts; their distribution and shape is fully compatible with the previously reported on SCID xenografts of MOLT-4 cells treated with CCL25^+^Wnt5a (42). This liver infiltration of tumor cells in MOLT-4 xenografts is, unlike in Jurkat xenografts, inhibited by the treatment with either 92R or Srb1, but not by isotype control mAbs. Kinetic analyses of MOLT-4 tumor cell liver infiltrations did not became detectable until day 21 after tumor cell injection, indicating that unlike the spleen and bone marrow that represent the initial colonizing organs for these tumor cells, the liver representes a secondary seeding organ. Importantly, 92R treatment started after the initiation of liver infiltrates (day 22), resulted in a 30% survival increase of the animals, suggesting that the treatment has a positive effect even at these later stages of tumor development. The ability of 92R (and Srb1) to inhibit infiltrates formation, is a relevant attribute of this Ab, related to its possible therapeutic use since, 30% to 70% of patients with advanced acute lymphoblastic leukemia before treatment develop infiltrates in the CNS (43). Finally, the data presented here also show the effects of 92R mAb on already formed infiltrates, demonstrating a significant decrease in the liver tumor cell numbers on orthotopic xenografts where the treatment started after day 25. In these experiments, two doses of mAb triggered a reduction of the number of tumor cells in the liver on the 92R-treated group, demonstrating the effectiveness of the mAb for the treatment of infiltrates already present in secondary seeded organs.

Finally, the antibody therapy showed a particularly striking benefit in combination with vincristine, a drug included in most contemporary clinical ALL treatment protocols. Indeed, suboptimal concentrations of Srb1 in combination with vincristine displayed a 5-fold synergistic effect between these drugs on survival of the xenotransplanted animals. This combination of drugs led to 40% of the animals to survive more than 400 days after tumor injection, where the experiment was discontinued. Interestingly, analysis of the surviving animals failed to detect tumor cells in the spleen, bone marrow and liver, demonstrating the robustness of the combination treatment.

Thus, this study addresses a direct clinical need in a disease difficult to treat, and documents preclinical proof of concept of the positive impact that CCR9-directed immunotherapy can have on the treatment of T-ALL and other CCR9 expressing malignancies. Suggesting that 92R and its humanized version (Srb1) represent promising candidates as therapeutic mAbs, either alone, or in combinations with other antibodies and/or chemotherapy, for precision medicine. The high relevance of the study relays on the clear clinical need for targeted therapies against T-ALL in general, and upon relapse in particular. One of the main caveats of targeted approaches for T-ALL is the sharing of antigenic targets between T-cells and T-ALL makes. However, CCR9 offers an attractive therapeutic target, as most circulating T-cells are CCR9^-^, and the expression of CCR9 is limited in normal tissues.

## Data availability statement

The original contributions presented in the study are included in the article/**Supplementary material**. Further inquiries can be directed to the corresponding authors.

## Ethics statement

The studies involving human participants were reviewed and approved by Bioethics Committee of the Consejo Superior de Investigaciones Cientificas and the medical ethics committee of the Hospital Universitario de La Princesa. The patients/participants provided their written informed consent to participate in this study. The animal study was reviewed and approved by Bioethics committee of the Consejo Superior de Investigaciones Cientificas and the Agriculture Department from the Community of Madrid with references PROEX 038/17 (to JAG-S) and PROEX 164/16 (to LK).

## Author contributions

SS and MD performed the *in vivo* experiments using the orthotopic T-ALL xenotransplant models, the subcutaneous xenotransplants using the AsPC-1 cells and the screening of different tumor cell lines for CCR9 expression. MB carried out the bioinformatics analyses of CCR9, determined the expression of CCR9 in the blood of T-ALL patients and in xenotransplants treated with 92R. EC generated the HEK293T-CCR9/GFP cell line and analyzed the xenotransplant experiment with these cells. IC-G and LK generated the HLPR primary T-ALL and performed the xenotransplant experiments with these cells. PL performed the *in vivo* experiments with MOLT-4-luc cells, the luciferase analyses and analyzed the survivors of **Figure 7**. MT provided several primary T-ALL cells and advice on the design of animal experiments. CM-C diagnosed the patients and provided the bone marrow cells. LK generated and characterized the 92R monoclonal antibody, provided all the 92R and isotype control antibodies for these experiments, and together with JG-S designed the research concept. SS, MD and JG-S were the main contributors to the manuscript writing. All the authors read, commented the manuscript and approved the final version.

## Funding

The work in the author’s laboratories was partially supported by grants from the PN2014-A from the ISCIII (PI14/00703, co-financed by FEDER funds from the EU, Operative program on Intelligent Growth 2014-2020, to LK); the Spanish Ministry of Economy, Industry and Competitiveness (RTC-2015-3786-1 to LK and JG-S), co-financed by FEDER funds from the EU and from the Spanish Ministry of Science and Innovation (PID2019-105404RB-I00 to JAGS and LK financed by MCIN/AEI/10.13039/501100011033). As well as the CSIC (PIE-201420E109 and PIE-201720E092, to LK).

## Acknowledgments

The authors would like to thank Ms. Sara Ortega for generating the MOLT4-GFP cells; E. Ramos and M. Llorente (CNB) for monoclonal antibody production, purification and characterization; Miguel A. Sánchez and María C. Ortiz from the CNB-Flow Cytometry Facility for help with cell analyses. Ms. O. Arbequilla and R. Sanchez (CIEMAT) for their help with cell sorting; the CIB-confocal unit for help with UV-stereomicroscopic images and confocal microscopy, and the CIB and CNB animal facilities for animal care and technical help. IC was hired with grants RTC-2015-3786-1 and PIE-201720E092 (both from LK). The humanized version of 92R (Srb1) was kindly provided by SunRock Biopharma, Ltd.

## Conflict of interest

92R mAb was generated by the lab of LK and developed by the labs of LK and JG-S and has been protected by the CSIC (US Patent 10,401,363; EP13382469.8); an additional patent application has been filed by the CSIC and SunRock Biopharma, Ltd. on the use of 92R in Srb1 in combination therapies for cancer (EP21382020.2). SunRock Biopharma, Ltd. (Santiago de Compostela, Spain) kindly provided the Srb1 antibody used for these experiments. Neither the financing bodies, the CSIC or SunRock Biopharma Ltd., had a role on the design of the study, data collection and analysis, nor in the decision to publish the manuscript. Therefore, the authors declare that the research was conducted in the absence of any commercial or financial relationships that could be construed as a potential conflict of interest.

## Publisher’s note

All claims expressed in this article are solely those of the authors and do not necessarily represent those of their affiliated organizations, or those of the publisher, the editors and the reviewers. Any product that may be evaluated in this article, or claim that may be made by its manufacturer, is not guaranteed or endorsed by the publisher.
