# Supplementary material for: Therapeutic potential of an anti-CCR9 mAb evidenced in xenografts of human CCR9+ tumors
Source: Front Immunol. 2022 Jul 27;13:825635. doi: 10.3389/fimmu.2022.825635 (PMC9363564; doi:10.3389/fimmu.2022.825635)
Supplement: Supplementary file 1 [file DataSheet_1.pdf]

Supplementary Figures

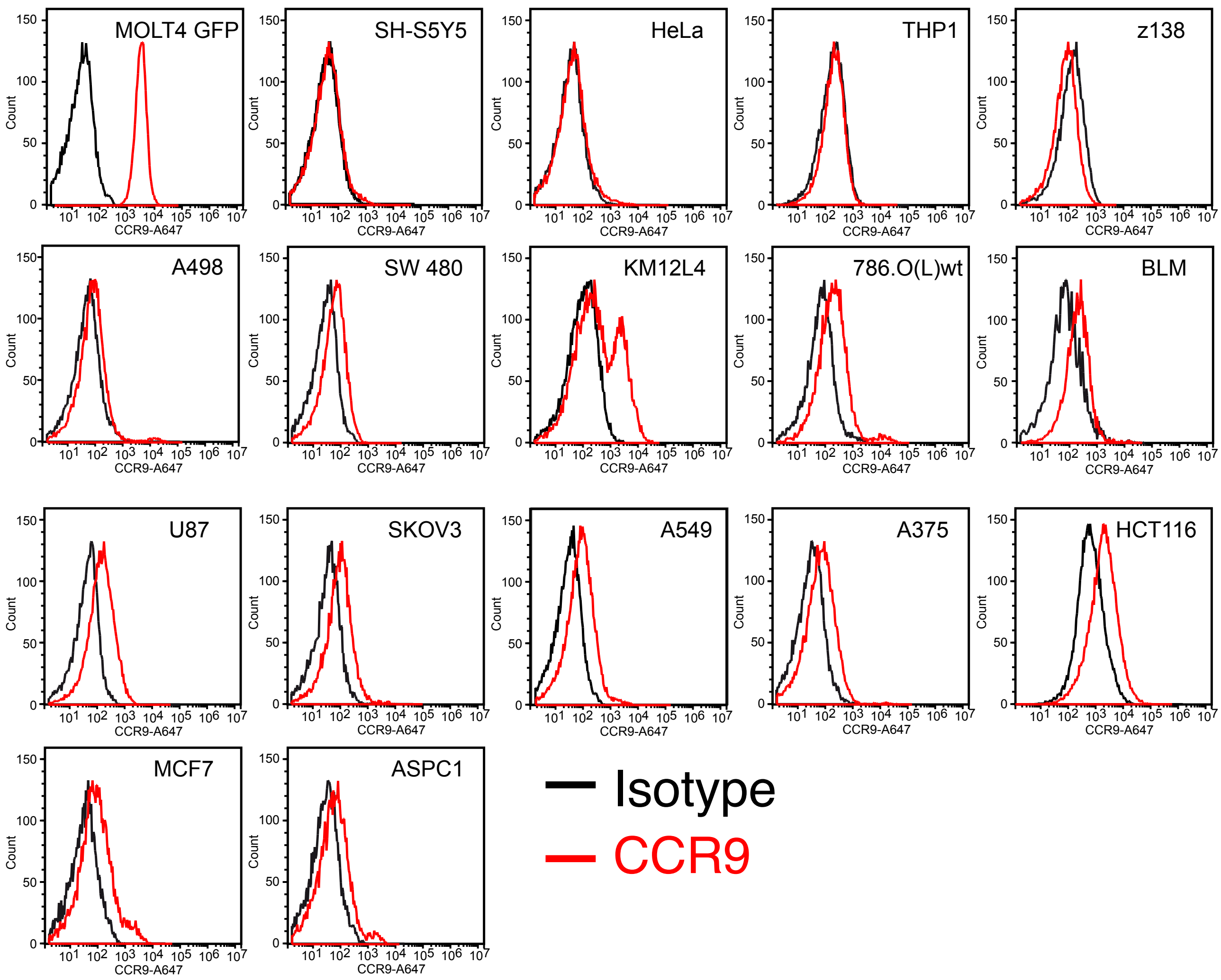

Supplementary Figure 1. CCR9 expression in different tumor cell lines from various origins. A panel of different tumor-derived cell lines were stained with either an isotype control antibody or the 92R mAb. The staining was revealed with an Alexa-647-goat anti-mouse IgG. The cytofluorimetric analyses were carried out gating on live cells. As positive control, MOLT4-GFP cells were used. The other cell lines analyzed were the neuroblastoma cell line SH-SY5Y (ATCC CRL-2266), the cervical carcinoma cell line HeLa (ATCC CRM-CCL-2), the acute monocytic leukemia cell line THP-1 (ATCC TIB-202), the mantle cell lymphoma Z-138 (ATCC CRL-3001), the kidney carcinoma cell lines A-498 (ATCC HTB-44) and 786-O (ATCC CRL-1932), the colorectal carcinoma cell lines SW480 (ATCC CCL-228) and KM12L4 (ATCC CCL-251), the melanoma cell lines A375 (ATCC CRL-1619) and BLM (RRID: CVCL_7035, Expasy Cellosaurus), the glioblastoma-derived cell line U87 (ATCC HTB-14), the ovarian adenocarcinoma cell line SKOV3 (ATCC HTB-77), the lung carcinoma cell line A549 (ATCC, CRM-CCL-185), the colon carcinoma HCT 116 (ATCC CCL-247), the mammary adenocarcinoma MCF7 (ATCC HTB-22) and the pancreas carcinoma AsPC-1 (ATCC CRL-1682)

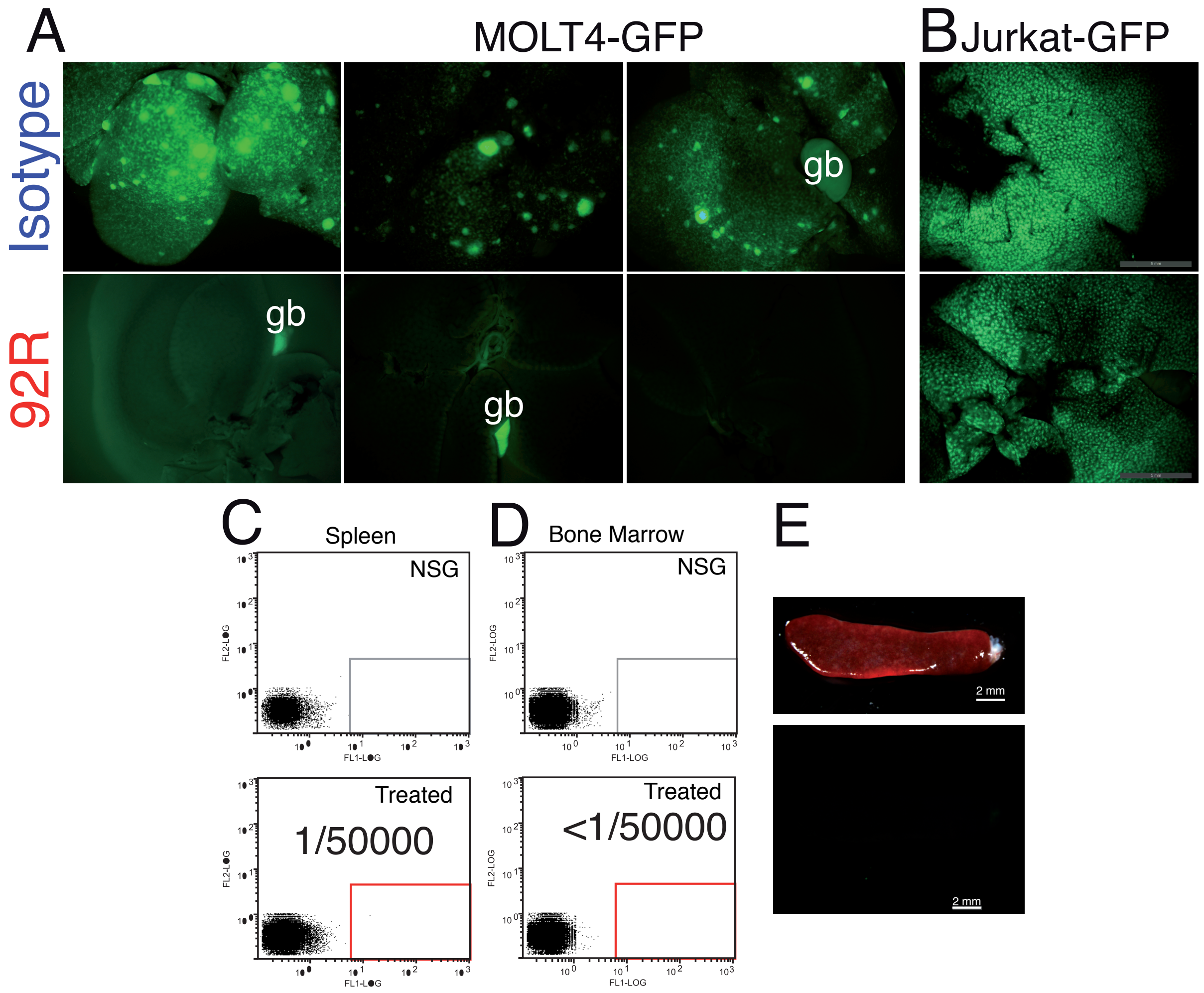

Supplementary Figure 2. Specificity of 92R mAb treatment on liver infiltrates and survival. (A) UV-stereomicroscopic images of three representative livers from NSG animals carrying MOLT4-GFP xenotransplants treated with either control isotype mAb (top row) or 92R mAb (low). The gallbladder (gb) is auto-fluorescent and visible in some of the livers. (B) UV-stereomicroscopic images of a representative liver from NSG animals carrying Jurkat-GFP xenotransplants treated with either isotype control mAb (top) or 92R mAb (bottom). (C,D,E) Analysis of a mouse, from Fig. 3C surviving 343 days after a MOLT4-GFP orthotopic xenotransplant treated with 92R mAb. Flow cytometry analyses of a single cell suspension from the spleen (C) and bone marrow (D of a wild-type NSG mouse (control) and of the animal that survived 343 days to the MOLT4-GFP orthotopic xenotransplant after treatment with 2 doses of 100 µg/dose of 92R mAb (Treated). On each sample, 50 000 live cells were analyzed. (E) Stereomicroscopic image of the spleen (top) and the corresponding UV image failing to detect GFP^+ cells (bottom)

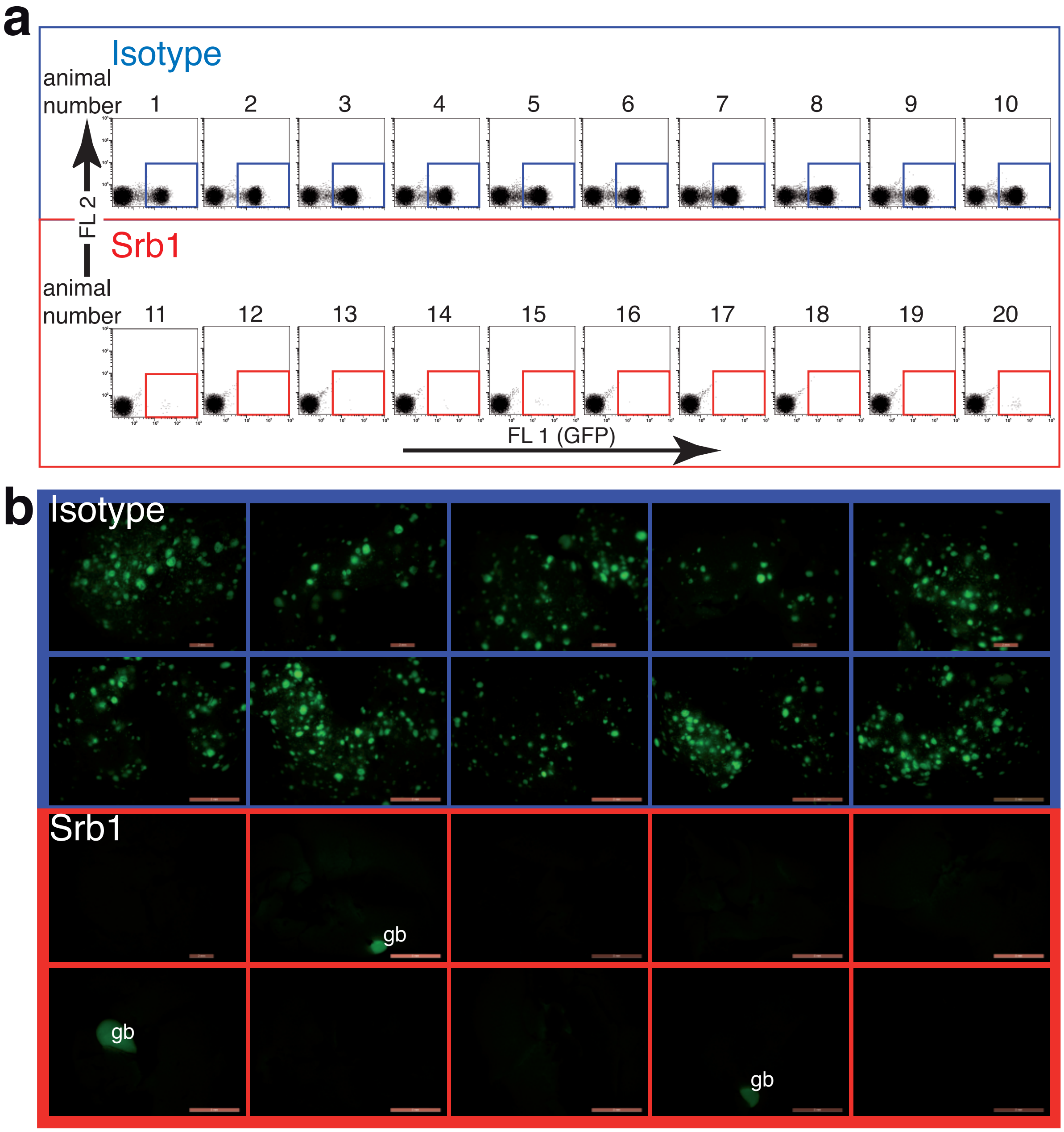

Supplementary Figure 3. Comparison of bone marrow and liver of xenotransplanted NSG mice after treatment with isotype control or Srb1 antibodies. The bone marrow and livers of the animals described in Fig. 4 were analyzed. (a) Flow cytometry analysis of the animals analyzed in Fig. 4 to determine the fraction of tumor cells (GFP^{+}) in the tissue. The results show that in the isotype control treated group between 16.39 and 63.13% of the cells are tumor cells, whereas on the Srb1-treated group, the tumor cells represented between 0 and 0.14% of the total cells. (b) UV-stereomicroscopic images of the livers from animals treated with isotype control mAb (top two rows) and from animals treated with Srb1 mAb (bottom two rows). The gallbladder (gb) is visible in some of the livers, due to its autofluorescence

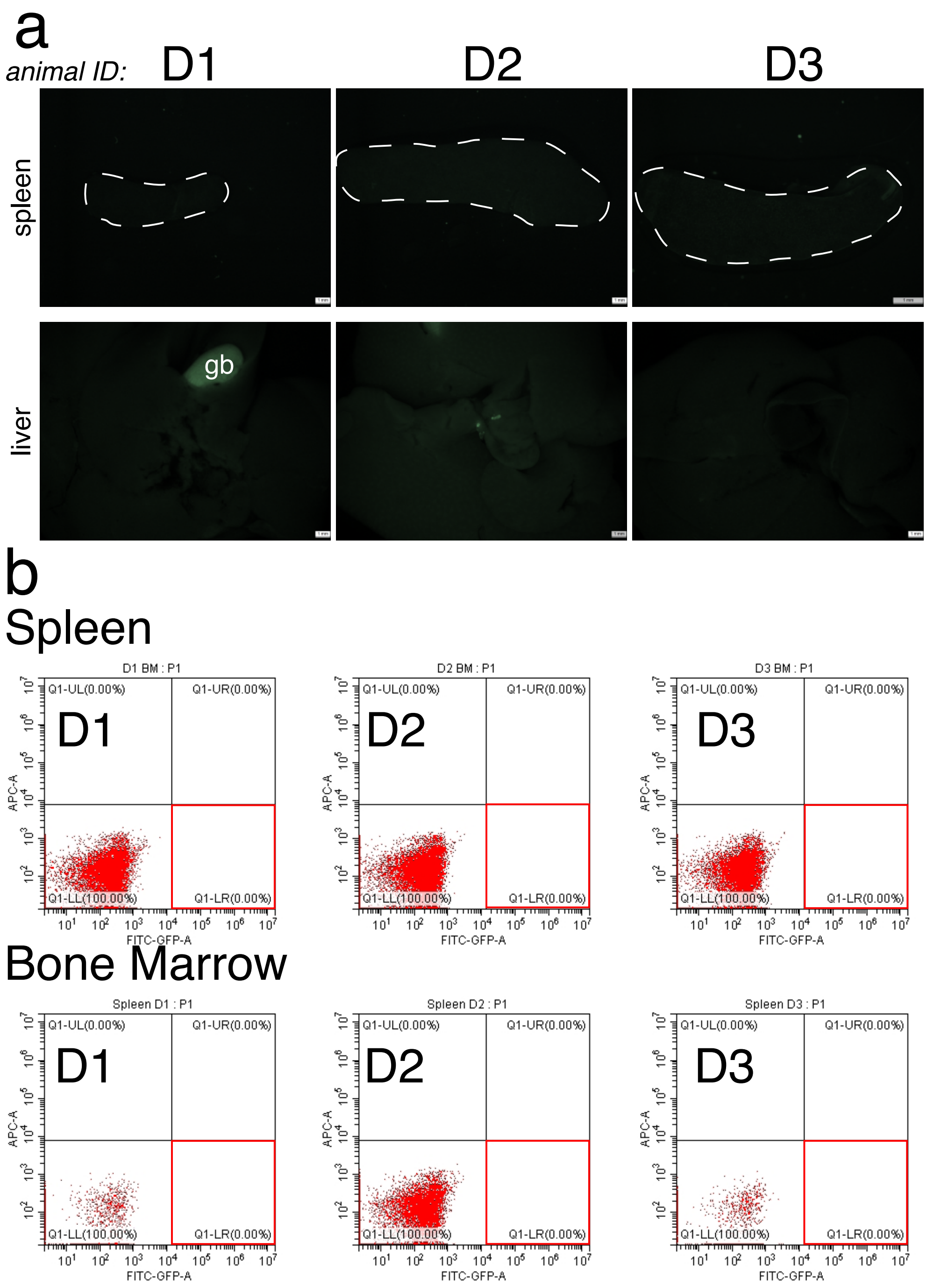

Supplementary Figure 4. Analysis of the animals censored in Fig 6. (a) UV-stereomicroscopic images of the three spleens (top) and livers (bottom) from NSG animals carrying MOLT4-GFP xenotransplants treated with a combination of vincristine and Srb1. The gallbladder (gb) is visible in one of the livers. (b) Flow cytometry analyses of the spleen (top) and bone marrow (bottom) from the same NSG animals demonstrating the lack of tumor cells in each of the analyzed samples (<1/50 000 cells), where 50 000 live cells were analyzed.
